# *In Vitro* Generation of Functional Liver Organoid-Like Structures Using Adult Human Cells

**DOI:** 10.1371/journal.pone.0139345

**Published:** 2015-10-21

**Authors:** Sarada Devi Ramachandran, Katharina Schirmer, Bernhard Münst, Stefan Heinz, Shahrouz Ghafoory, Stefan Wölfl, Katja Simon-Keller, Alexander Marx, Cristina Ionica Øie, Matthias P. Ebert, Heike Walles, Joris Braspenning, Katja Breitkopf-Heinlein

**Affiliations:** 1 Medicyte GmbH, Im Neuenheimer Feld 581, 69120, Heidelberg, Germany; 2 Department of Medicine II, Faculty of Medicine at Mannheim, Heidelberg University, Mannheim, Germany; 3 upcyte technologies GmbH, Osterfeldstraße 12–14, 22529, Hamburg, Germany; 4 Institute of Pharmacy and Molecular Biotechnology, Heidelberg University, Heidelberg, Germany; 5 Institute of Pathology, University Medical Centre Mannheim, Heidelberg University, Mannheim, Germany; 6 Vascular Biology Research Group, Department of Medical Biology, Faculty of Health Sciences, University of Tromsø, Sykehusgt. 44, N–9037, Tromsø, Norway; 7 Tissue Engineering and Regenerative Medicine, University Wuerzburg, Roentgenring 11, 97070, Würzburg, Germany; University of Navarra School of Medicine and Center for Applied Medical Research (CIMA), SPAIN

## Abstract

In this study we used differentiated adult human upcyte^®^ cells for the *in vitro* generation of liver organoids. Upcyte^®^ cells are genetically engineered cell strains derived from primary human cells by lenti-viral transduction of genes or gene combinations inducing transient proliferation capacity (upcyte^®^ process). Proliferating upcyte^®^ cells undergo a finite number of cell divisions, i.e., 20 to 40 population doublings, but upon withdrawal of proliferation stimulating factors, they regain most of the cell specific characteristics of primary cells. When a defined mixture of differentiated human upcyte^®^ cells (hepatocytes, liver sinusoidal endothelial cells (LSECs) and mesenchymal stem cells (MSCs)) was cultured *in vitro* on a thick layer of Matrigel™, they self-organized to form liver organoid-like structures within 24 hours. When further cultured for 10 days in a bioreactor, these liver organoids show typical functional characteristics of liver parenchyma including activity of cytochromes P450, CYP3A4, CYP2B6 and CYP2C9 as well as mRNA expression of several marker genes and other enzymes. In summary, we hereby describe that 3D functional hepatic structures composed of primary human cell strains can be generated *in vitro*. They can be cultured for a prolonged period of time and are potentially useful *ex vivo* models to study liver functions.

## Introduction

The idea to construct *in vitro* 3D tissue-like structures to be used as model system for the respective organ is an appealing experimental approach. The main focus hereby is to exploit the *in vivo* physiological mechanism that occurs during organ development or healing (regeneration) and to implement similar mechanisms to develop a functional tissue *in vitro*. Such 3D liver-like structures would for example meet the needs of the pharmacological and toxicological industry for drug screening [1]. The main techniques to generate 3D cellular constructs are either the formation of spheroids or building of tissue-like structures by placing sheets of cells and extracellular matrix components on top of each other (reviewed in [2]). The disadvantage of spheroids is that the cells are distributed randomly without formation of spatial organization i.e., liver spheroids neither possess typical hepatic cord-like alignment of polarized hepatocytes nor sinusoids lined with endothelial cells reflecting the *in vivo* situation. Similarly, 3D liver models generated using sandwich cultures can never fully recapitulate the true *in vivo* architecture of the organ. Such *ex vivo* formation of tissue for most complex organs such as heart, kidney or brain would be very challenging. However, the liver is exceptional in its ability to regenerate. It is well established that fully differentiated adult liver is capable of regeneration as long as a sufficient amount of intact liver remains after damage (reviewed in [3] and [4]). Therefore, in principle any differentiated adult liver cell should harbor the potential to proliferate and regenerate to a complex and functional organ under suitable conditions. Indeed, Takebe and colleagues described recently that induced-pluripotent stem cells (iPSC) stimulated to become hepatic endoderm-like cells (iPSC-HE) together with mesenchymal stem cells (MSC) and human umbilical vein endothelial cells (HUVEC), self-organized *in vitro* into macroscopically visible 3D cell clusters by an intrinsic organizing capacity [5]. When these structures were transplanted into mice, they became vascularized, engrafted into the recipient’s tissue and produced hepatic factors like albumin. Possible applications of such an organoid structure include replacement therapy but also the possibility to study hepatotoxic effects of new compounds. They could as well be used as simplified model system to investigate processes like liver regeneration, fibrogenesis or malignant transformation. Due to the fact that these organoids are formed out of different cell types, which are in 3D contact to each other, they can be expected to represent a system which is much closer to the depicted *in vivo* situation than conventional approaches.

In the present work we analyzed if liver organoids could also be generated from adult, differentiated cells and if these organoids can be cultured for long-term to study liver functions. Instead of stem cells, upcyte^®^ hepatocytes were used to reflect the parenchymal cells of the liver. In order to depict the native physiological condition of liver, we further used upcyte^®^ liver sinusoidal endothelial cells (LSECs) instead of conventional endothelial cells like HUVECs. LSECs are a specialized type of scavenger endothelial cells that are able to endocytose an array of physiological and foreign macromolecules and colloids from the blood [6]. The upcyte^®^ process involves stable transduction of primary cells with lentiviral constructs carrying sequences which code for certain proliferation-inducing factors. These cells are cultured in medium containing a defined mixture of growth factors, allowing tighter control over proliferation (up to 40 population doublings) [7], [8]. Employing this process, almost unlimited numbers of cells from one donor can be obtained. The performance of upcyte® hepatocytes compared to primary cells has been extensively studied and it was demonstrated that these cells are highly similar to primary cells [9], [10].

Our results show that by using upcyte^®^ cells, liver organoids can be generated and these organoids after culturing them for a period of 10 days, express several marker proteins, genes and enzymes to a degree that is comparable to adult human liver. Furthermore, the architecture of these liver organoids to some degree resembles typical hepatic structures. Potential future applications of such *in vitro* generated hepatic organoids include pre-clinical drug screening or investigation of liver injury processes like fibrogenesis, cirrhosis or inflammation prior to animal studies, or even as a replacement for animal studies.

## Materials and Methods

### Materials

Human upcyte^®^ hepatocytes, upcyte^®^ LSECs and upcyte^®^ MSCs, upcyte^®^ Hepatocyte Growth Medium and upcyte^®^ High Performance Medium, upcyte^®^ LSEC Medium were from Medicyte GmbH, Heidelberg, Germany. Liver tissue was obtained from Tissue Solutions, Glasgow, UK. The tissue provider holds the required ethics approvals in place, and ensures that it has copies of this and their patient consent forms. Copies of sources Ethics approval and template donor consent forms are available on request. Foetal bovine serum was obtained from PAN GmbH, Aidenbach, Germany. BD Matrigel™ Basement Membrane Matrix was from Corning. Testosterone, 6β-hydroxytestosterone, and 4′,6-diamidino-2-phenylindole (DAPI) were from Sigma-Aldrich, Germany. CD31 antibodies were from DAKO, Eching, Germany. Mouse anti-Ck8 (C51), mouse anti-Ck18 (DC–10) and vimentin (E–5) were from Santa Cruz Biotechnology, Heidelberg, Germany. Rabbit anti-albumin from Sigma, Mouse anti-E-Cadherin from BD Transduction Laboratories, Alexa Fluor 546 goat anti-rabbit and Alexa Fluor 488 goat anti-mouse from Invitrogen were used for immunofluorescence staining. Nuclei were counterstained with Draq5 from BioStatus. Cy3 goat anti-mouse IgG was from Dianova, Hamburg, Germany. All other chemicals and media are either mentioned in the corresponding text below or were obtained from standard commercial suppliers. The primary human hepatocytes used for the upcyte® process (see below) were initially purchased from Cellzdirect/Life Technologies.

### Cell culture and liver organoid formation

The upcyte^®^ process was performed according to Burkard et al., 2012 [8] and Levy et al. [9]. Cells were cultured at 37°C, 5% CO_2_ and 95% humidity. The cells were passaged at 70–80% confluence and living cells were counted using Trypan Blue exclusion. For the generation of liver organoids, required culture plates, bioreactors and pipette tips were pre-cooled at 4°C before use and Matrigel^™^ was thawed overnight at 4°C. Matrigel™ was diluted 1:1 with upcyte^®^ LSEC Growth Medium. For generation of one liver organoid in a 24-well format or in the bioreactors, 380 μl of diluted Matrigel™ (~200 μl/cm^2^) was added to the plates/chambers and incubated at 37°C for 30 to 45 minutes to allow for polymerization. Trypsinized cells were re-suspended in liver organoid growth medium (upcyte^®^ Hepatocyte Growth Medium and upcyte^®^ LSEC Growth Medium in 1:1 ratio). 1.0 x10^6^ upcyte^®^ hepatocytes (from the Donor No. 422A-03), 1.0 x10^6^ upcyte^®^ LSECs and 0.2 x10^6^ upcyte^®^ MSCs were mixed in 1 ml of liver organoid growth medium, added to the Matrigel™-coated plates/chambers and further incubated at 37°C, 5% CO_2_ and 95% humidity for the formation of liver organoids. We tested two setups of bio-reactors, the Quasi-vivo^®^ System from Kirkstall and the Live-box 1 system, developed by the group of Prof. Arti Ahluwalia, Faculty of Engineering University of Pisa, Italy (IVTech srl, Lucca, Italy).

### *In situ* hybridization

Detection of mRNA transcripts in paraffin sections was performed as described previously [11],[12]. The protocol is based on the preparation of double-stranded DNA *in vitro* transcription templates using polymerase chain reaction (PCR), with primers that include RNA polymerase promoter sequences and size-based purification of PCR fragments containing the target gene-specific cDNA and promoter elements for T7 and SP6 RNA polymerase. The primer sequences used are listed in Table 1.

**Table 1:** Gene specific sequences of the primers used for in situ hybridizations.

Gene name	NCBI Ref. Seq.	Forward	Reverse
KRT18	NM_000224.2	GGTCAGAGACTGGA-GCCATTACTT	CCAGCTTGACCTTGAT-GTTCAGCAG
Albumin	NM_000477.5	GGTGAGACCAGAGG-TTGATGTGATG	CACACATAACTGGTTC-AGGACCACG
Glutaminase2	NM_013267.2	GTGTGTGAGCAGCA-ACATTGTGCTC	GATGGCTCCTGATACA-GCTGACTTG
Glutamine synthetase	NM_001033044.2	GTTGCCTGAGTGGA-ATTTCGATGGC	CGGTTTCATTGAGAAG-ACACGTGCG
HIF1α	NM_001243084.1	CATGGAAGGTATTG-CACTGCACAGG	CAGCACTACTTCGAAG-TGGCTTTGG
G6P	NM_000151.2	GTGGCGTATCATGC-AAGTGCTATGC	GAGGCTGAGACATGA-GAATCGCTTG

### CYP assay

For measurement of CYP activities (CYP2B6, 2C9 and 3A4), the liver organoids cultured for 10 days were incubated with each of the substrate separately. The substrates used were bupropion-HCl, tolbutamide and testosterone to assess CYP2B6, 2C9 and 3A4 activities respectively. The samples were transferred to a fresh 96-well plate and processed for HPLC analysis according to Burkhard et al. [8]. The metabolites were analysed using UV-HPLC. For tolbutamide, bupropion and their metabolites, 0.1 mg/ml chlorpropamide was used as internal standard. For testosterone and its metabolite, 0.5 mM cortexolone was used as internal standard. The mobile phases used for tolbutamide, bupropion and its metabolites were (A) 1.36 g KH_2_PO_4_ in 800 ml cell culture grade distilled water made up to 1 l (pH 4.6), 52.6 ml 100% acetonitrile and (B) 500:500 acetonitrile:water. The mobile phases for testosterone and its metabolites were (A) 390:600:10 methanol:water:acetonitrile and (B) 800:180:20 methanol:water:acetonitrile. All the metabolites and its respective internal standard were separated on a SunFire C18 2.5 μm 2.1×20 mm column (Waters, Munich, Germany). The peaks were detected on an UV detector set to 200/229 nm for tolbutamide, bupropion and 252 nm for testosterone.

### Immunocytochemistry

Cells were first fixed with 4% PFA for 5 min at RT, then rehydrated for 60 min using 3% bovine serum albumin (BSA) in PBS. Cells were incubated for 45 min with the primary antibody diluted in 0.2% BSA/PBS to detect CD31 (1:300), vimentin (1:200) or cytokeratin 8/18 (1:50). After washing, the secondary antibodies Cy3™- conjugated AffiniPure goat anti-mouse IgG (1:200, Jackson Immuno Research, Baltimore, USA) diluted (1:200) in 0.2% BSA/PBS containing DAPI (final conc. 300 nM) were incubated for 45 min at 37°C. Finally, the samples were washed three times with 0.2% BSA/PBS and observed using phase contrast microscopy.

### Immunohistochemistry

Immunoperoxidase-based immunohistochemistry was performed to stain 2 μm sections of paraffin-embedded liver organoids. The following primary antibodies were used: mouse anti human CK8/18 (monoclonal, concentrated, pH6; BD Pharmingen, San Jose, CA, USA), rabbit anti human Vimentin (monoclonal, 1:400, pH6; Thermo scientific, Waltham, MA, USA), mouse anti human CD31 (monoclonal, 1:500, pH9; DAKO), mouse anti human E-Cadherin (monoclonal, 1:50, pH9, Thermo scientific), mouse anti human Ki67 (monoclonal, 1:800, pH6, DAKO); Immunohistochemistry was performed as described in detail elsewhere [13] using the following chemicals and reagents: antigen retrieval in Novocastra antigen retrieval solution pH6 or pH 9.0 (Leica, Wetzlar, Germany); blocking of endogenous peroxidase (DAKO blocking solution, DAKO, Hamburg, Germany); detection of bound antibodies by the immunoperoxidase/DAB-based DAKO REAL detection system (DAKO). For immunofluorescence staining, liver organoid sections were incubated overnight (37°C) with primary antibodies, mouse anti-E-Cadherin (1:100) and rabbit anti-albumin (1:500) or non-immune IgGs diluted in blocking buffer, washed and incubated with secondary antibodies in blocking buffer for 1 h at room temperature. Nuclear stain was Draq5 (diluted 1:1000 in PBS). Sections were analysed in a Zeiss LSM 510 confocal laser scanning microscope (Carl Zeiss, Germany).

### RNA isolation and real-time PCR

Total RNA was isolated from liver organoids using “high pure RNA isolation kits” from Roche including on column genomic DNA digestion with RNase free DNase (Mannheim, Germany). 1 μg total RNA was reverse transcribed to cDNA using the "Transcriptor First Strand cDNA Synthesis Kit from Roche. For quantitative real-time PCR (using SyberGreen) for the detection of expressions of drug metabolizing enzymes "Prime PCR assays and controls" from BioRad (München, Germany) were used. For the detection of albumin, E-Cadherin and ZO–1 the following primers were used: ZO–1 F: 5'-ACCAGTAAGTCGTCCTGATCC–3'; R: 5'-TCGGCCAAATCTTCTCACTCC–3'; E-Cadherin F: 5'-TGAAGGTGACAGAGCCTCTGGAT–3'; R: 5'-TGGGTGAATTCGGGCTTGTT–3'; Albumin F: 5'-CTTGAATGTGCTGATGACAGG–3'; R: 5'-GCAAGTCAGCAGGCATCTCAT–3'. Data were analyzed using the ΔCt method and expression values were normalized to the expression levels of the house-keeping gene HPRT1 as indicated.

## Results

### Spontaneous self-assembly of liver organoids using upcyte® cells

**Fig 1:**
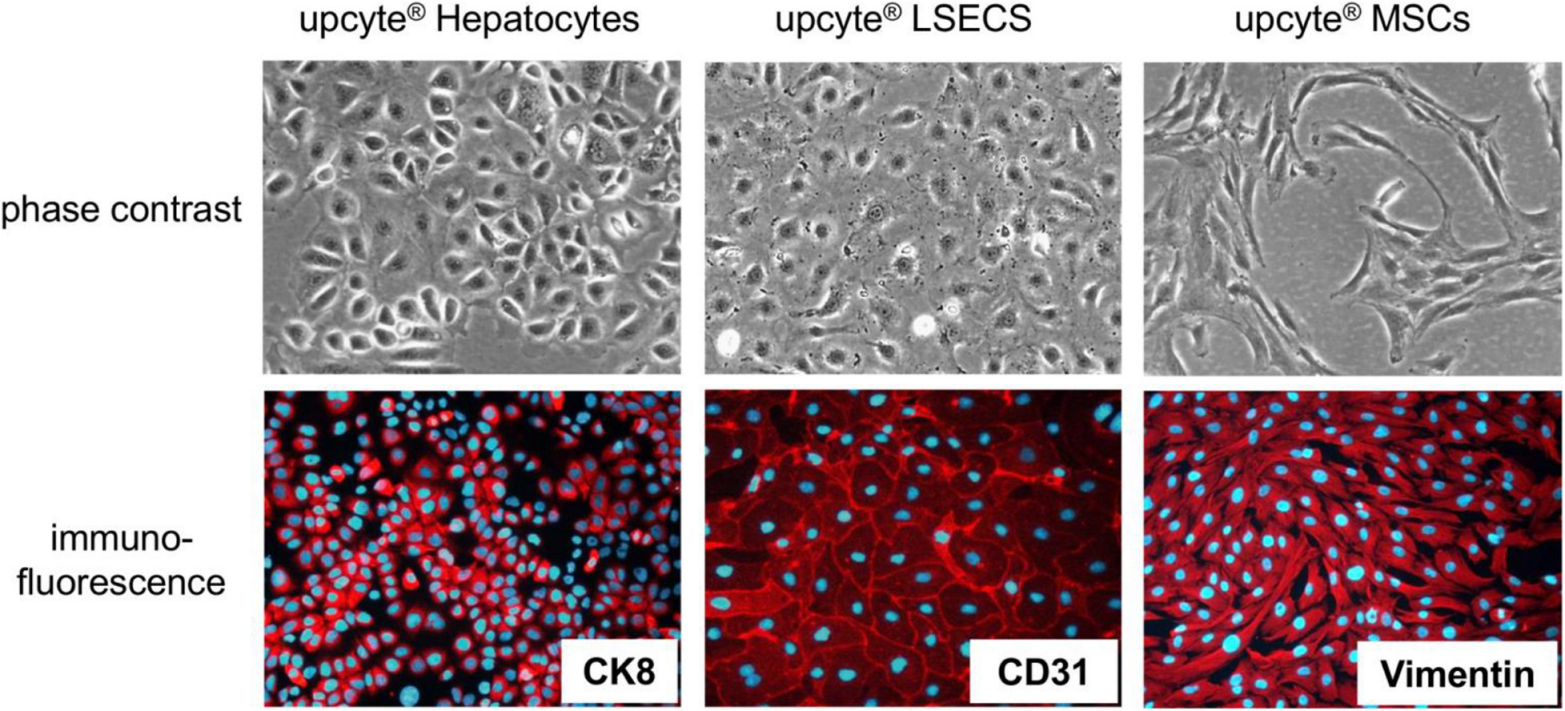
Appearance of the cells used for organoid formation. Phase contrast photographs and immuno-fluorescent stainings for specific cell type markers in the three different types of upcyte^®^ cells which were used for liver organoid generation. CK: Cytokeratin.

In order to generate liver organoids we used three different upcyte® cell types (all human): hepatocytes, liver sinusoidal endothelial cells (LSECs) and mesenchymal stem cells (MSCs). Fig 1 shows the typical morphology of these cells in monolayer culture and the presence of cell-type specific marker proteins.

**Fig 2:**
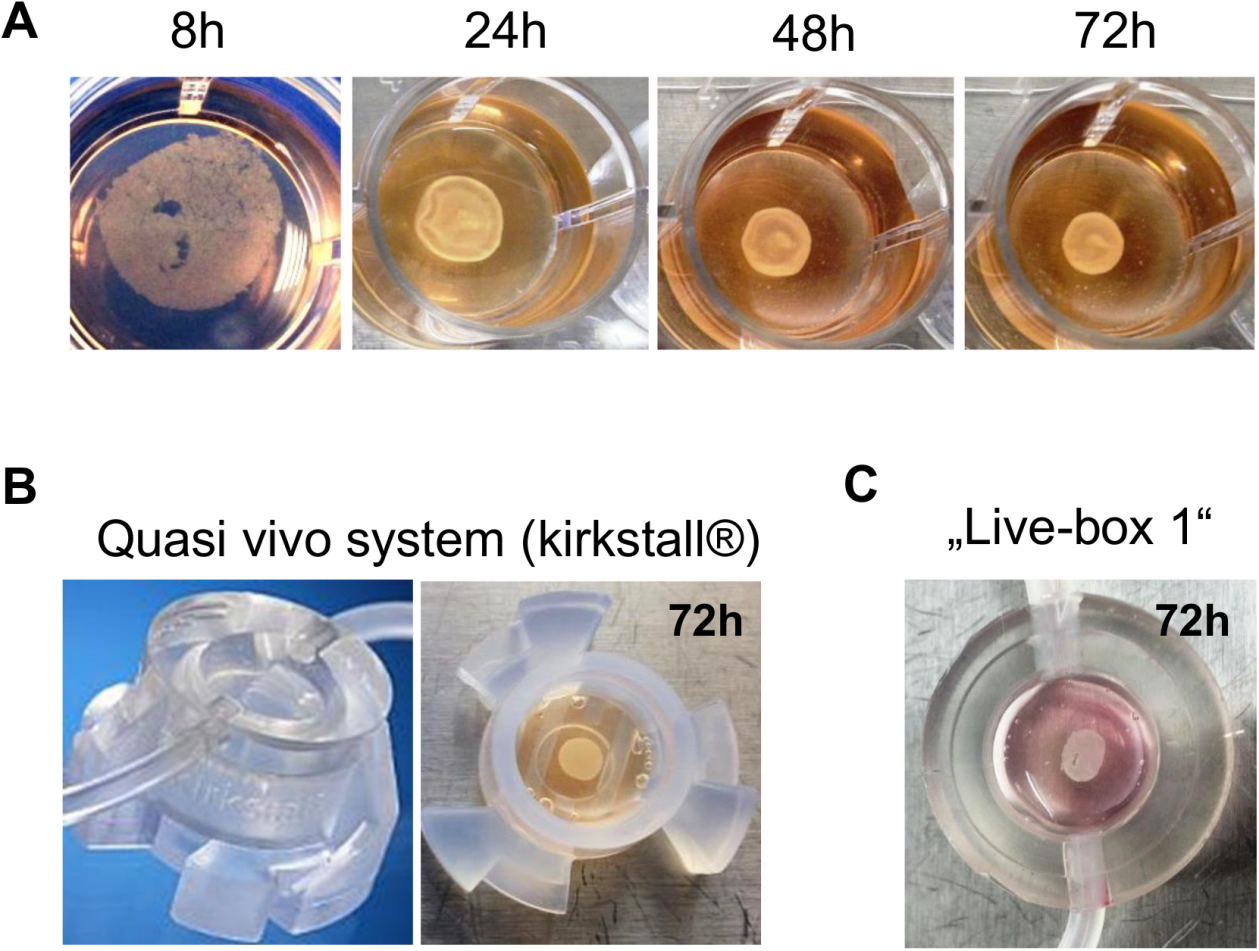
Generation of liver organoids *in vitro*. (A) Time-dependent formation of liver organoids in 24-well plates. Long-term culture (>48h) was performed in a dynamic system, e.g. the quasi vivo system from kirkstall^®^ (B) or the Live-box 1 system, generated by the group of Prof. Arti Ahluwalia, Faculty of Engineering, University of Pisa, Italy (C).

When plated on Matrigel™-coated 24-well plates, defined mixtures of these three cell types self-assembled into a compact organoid structure (Fig 2A). In order to test if this process can be downscaled to formats commonly used in industrial settings, i.e. 48 and 96 well plates, cell number, medium and Matrigel™ volumes were reduced according to the surface area, which still resulted in formation of organoids (S1 Fig). To avoid any nutrient or oxygen-deficiency, liver organoids generated under static conditions (in the bioreactors) were connected to a dynamic system after 24 h and constantly perfused with medium for 10 days. We tested two setups of bio-reactors, the Quasi-vivo^®^ System from Kirkstall and the Livebox 1 system, developed by the group of Prof. Arti Ahluwalia, Faculty of Engineering University of Pisa, Italy (IVTech srl, Lucca, Italy) (Fig 2B and 2C). In both systems macroscopically similar liver organoids formed within a comparable time. H&E staining of samples from both systems were compared and in both no strong necrosis was observed (in contrast to static conditions for more than 72h, data not shown). For all further experiments the Kirkstall system was used.

### Spatial distribution of the diverse cell types within the organoids

**Fig 3:**
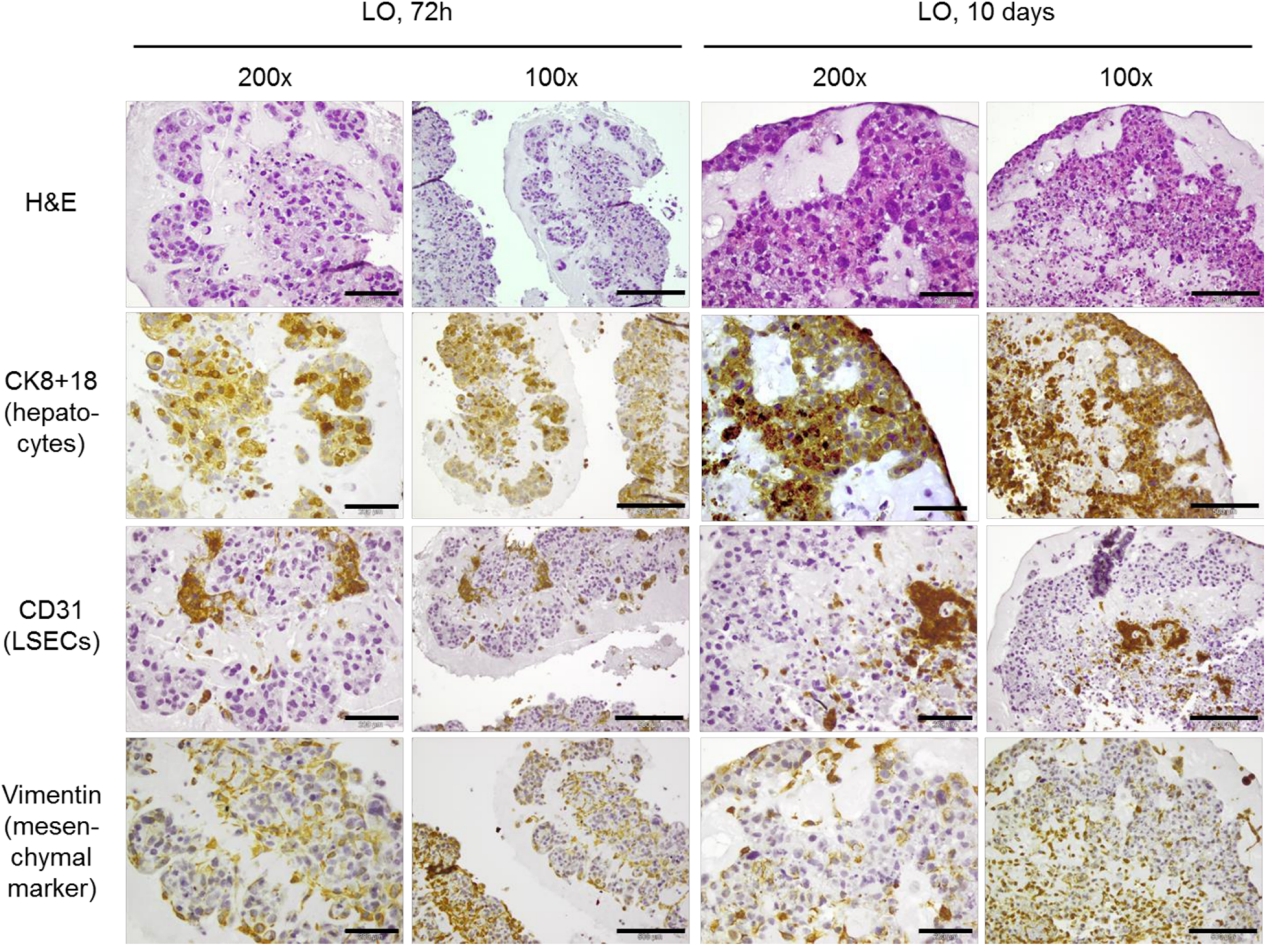
Spatial distribution of the different cell types within the liver organoids. H&E- and immuno-stainings for cell-type markers were performed in serial sections of liver organoids (LO) after 72h and 10 days of culture. The scale bars for the original magnification of 200x equals 200 μm, that of the original magnification of 100x equals 500 μm.

In order to determine the individual localization of the three different cell types at early and late stages of organoid formation, immunohistochemical stainings using antibodies against cell-type specific marker proteins were performed (Fig 3). The results demonstrate that the different cell types are not simply randomly distributed within the organoid but that they rapidly start to form structures with reproducible spatial distribution of hepatocytes and LSECs. Upcyte^®^ hepatocytes arranged in closely adherent clusters and moved towards the periphery of the liver organoid forming parenchymal areas while CD31-positive upcyte^®^ LSECs were mainly localized at the central regions. By contrast, MSCs (stained for vimentin) were distributed randomly between hepatocytes and LSECs.

### Hepatocytes inside the organoid express mRNA for typical markers as well as for many hepatic enzymes and they show typical liver morphology

**Fig 4:**
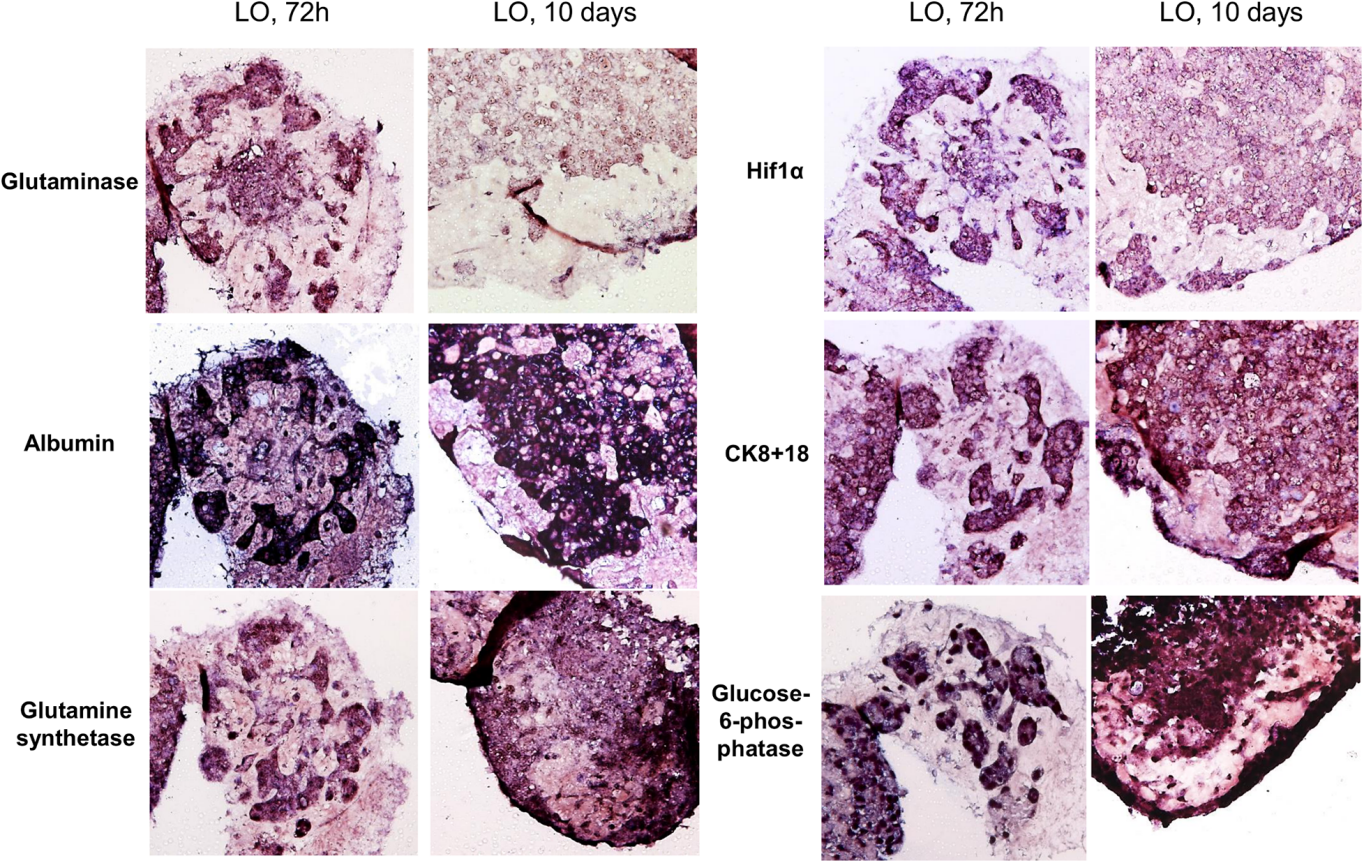
Expression of functional genes and hepatocyte markers on the RNA level. mRNA transcripts of the indicated genes were detected by *in situ* hybridization in serial sections of liver organoids (LO) after 72h and 10 days of culture.

**Fig 5:**
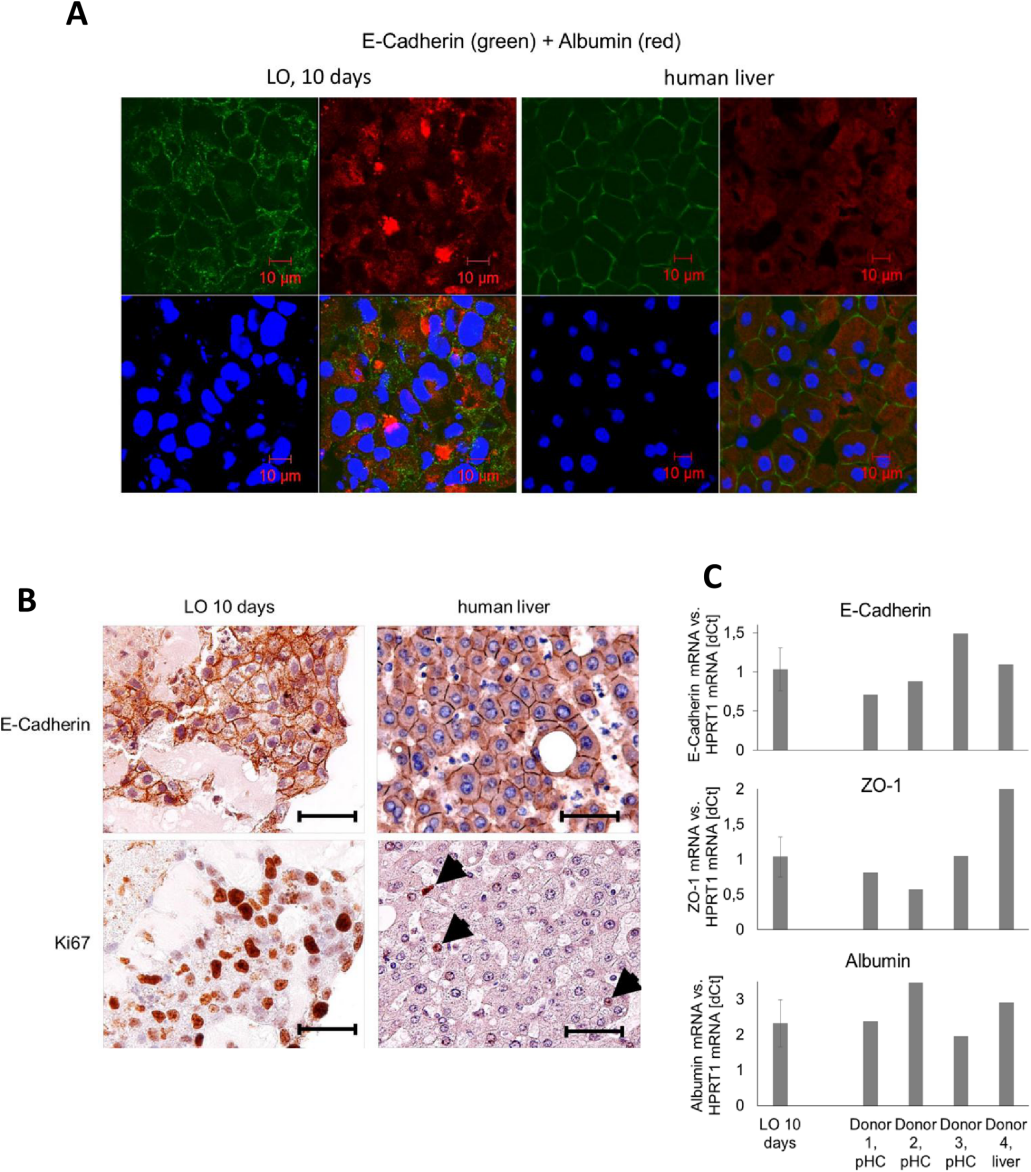
Immunostainings of a 10-day liver organoid in comparison with adult human liver. **A)** Immunofluorescent staining for the epithelial marker protein E-Cadherin (green) and the functional marker protein albumin (red) in a 10-day LO on the left and a cross-section of adult human liver on the right. The scale bar equals 10 μm. **B)** Immunohistochemical stainings for E-Cadherin and the proliferation marker Ki67 in a 10-day liver organoid (left panel). Human liver sections were used as control (right panel). The arrows point to Ki67-positive cells in the liver. Scale bars equal 100 μm (original magnification: 400x). **C)** The expression levels of the hepatocyte polarization markers E-Cadherin and ZO–1 as well as albumin were further analyzed by real-time PCR in samples from 10-day LOs (the average value of 4 different LOs +/-SD is shown). For comparison samples from four different donors were analyzed in parallel. Expression of the house-keeping gene HPRT–1 was used for normalization. pHC = primary hepatocytes (non-cultured).

In an attempt to further characterize the functional status of the hepatocytes within the organoids, we performed *in situ* hybridizations on serial paraffin sections of organoids from early and late stages. The results demonstrate that hepatocytes within the organoid retain a constitutive expression level of typical markers (e.g. cytokeratin 8 and 18) as well as important functional hepatic genes like albumin or enzymes of the glutamine and glucose-metabolism (Fig 4). To further investigate the functionality and polarity of the hepatocytes within the organoids immunofluorescent double-staining of the epithelial marker protein E-Cadherin and albumin was performed in 10-day liver organoids and was directly compared to adult human liver (Fig 5A). The presence and membraneous localization of E-Cadherin was further confirmed by immunohistochemistry (Fig 5B). On the mRNA level expressions of E-Cadherin, albumin and the cell-cell contact protein zona occludens 1 (ZO–1) were analyzed by real-time PCR and compared to primary human hepatocytes or whole liver from a total of four different donors (Fig 5C). In adult, differentiated liver cell proliferation is a rare event (see arrows in Fig 5B, lower right picture). In 10-day liver organoids in contrast we found strong positive staining for the proliferation marker Ki67 (Fig 5B, lower left picture) indicating that such organoids did not yet reach a fully differentiated, quiescent status. In summary these data indicate that at least some portion of the hepatocytes within the liver organoids express polarity markers (E-Cadherin and ZO–1) and synthesize albumin to a degree which is comparable to adult human liver or primary human hepatocytes.

### Hepatocytes inside the organoids express drug metabolizing enzymes

**Fig 6:**
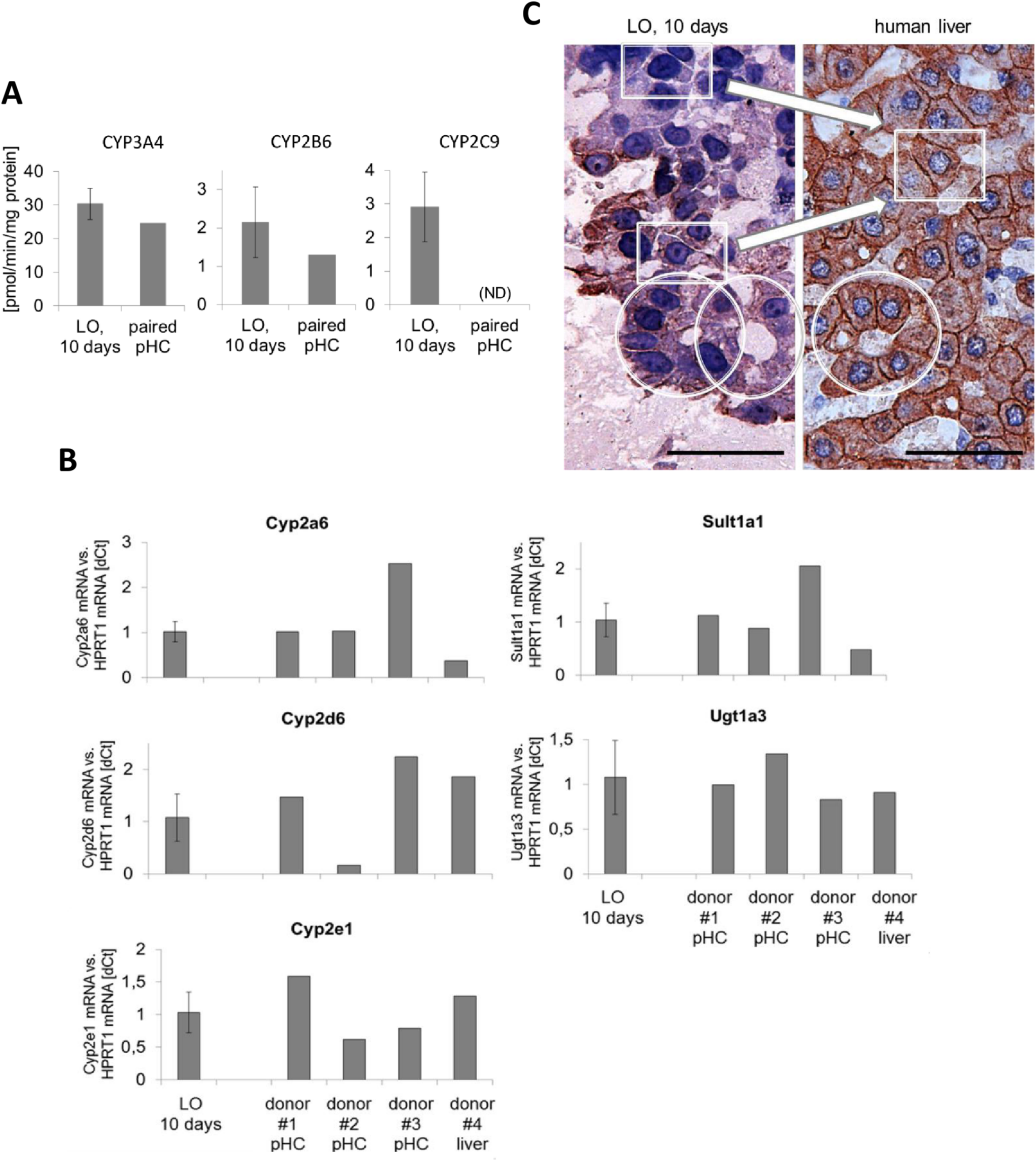
Expression and activity of drug metabolizing enzymes. Comparison of 10-day LOs with human liver. **A)** The basal activities of Cyps 3A4, 2B6 and 2C9 were measured (average of n = 4 +/-SD) in 10-day liver organoids (LOs). LOs were formed as shown in Fig 2 inside a bioreactor (Quasi-vivo^®^ System from Kirkstall). Substrate incubation was performed for 60 min on day 10 of culture. CYP-activity was normalized to total protein content of the organoids. Paired pHC are the same primary hepatocytes which had been upcyted® and used to form the organoids. Basal CYP activity levels of primary cells shown in the figure were provided by the manufacturer. For CYP2C9 no value was given (ND, not determined). **B)** Three more CYPs as well as Sult1a1 and Ugt1a3 were investigated on the mRNA expression level by real-time PCR. The average values of 4 different LOs +/-SD is shown. For comparison RNA samples from 4 donors were analyzed in parallel. The samples from donor 1–3 were RNAs from hepatocytes which were isolated, frozen down without primary culture and directly lysed after thawing (pHC; non-cultured cells). The sample from donor 4 represents the total RNA isolated from a piece of whole liver. Expression of the house-keeping gene HPRT–1 was used for normalization. **C)** Cross-sections of a 10-day LO (on the left) in direct comparison to adult human liver (on the right; stained against cytokeratins plus haematoxylin). The rectangles mark representative cells showing the typical cuboidal shape of polarized hepatocytes. The circles highlight areas where hepatocytes have arranged around circular openings. The scale bar equals 100 μm.

To further investigate if hepatocytes show functional properties of liver parenchymal cells, liver organoids were analyzed for basal activity of three prototypic CYPs, CYP-3A4, -2B6 and 2C9. CYP3A4 is an enzyme that metabolizes a large number of clinically important substrates [14]. Drugs metabolized mainly by CYP2B6 include artemisinin, bupropion, cyclophosphamide, efavirenz, ketamine, and methadone [15] while CYP2C9 is the principal isoform of the CYP2C subfamily in the human liver and is involved in the oxidation of several endogenous and xenobiotic compounds, including many therapeutic drugs [16]. The results show that hepatocytes within the liver organoids produce all these 3 enzymes. The basal activity levels were very comparable to that of the primary human hepatocytes which were initially used to generate the upcyte® hepatocyte strain (Fig 6A). On the mRNA level expressions of five additional drug metabolizing enzymes (CYPs 2a6, 2d6, 2e1 as well as Sult1a1 and Ugt1a3) were determined by real-time PCR and were compared to primary human hepatocytes or whole liver from 4 different donors (Fig 6B). While there was an expectable variability in the expression levels between the four donors the levels of expression in the 10-day organoids was mostly within an average range. This further supports the conclusion that hepatocytes within the organoids are functional cells allowing the use of these structures to analyze e.g. hepatic toxicity of certain substances or drugs. Comparing the overall architecture of the parenchymal areas within the LO's with adult human liver we noticed that the cell shape of individual hepatocytes frequently resembled that of primary cells in the liver (see rectangular frames in Fig 6C) and hepatocytes often formed circular arrangements of cells, also resembling similar structures of adult liver. However, the average seize of the nuclei and the relation of nucleus to cytoplasm of the LO-hepatocytes is different from the adult liver with nuclei being frequently bigger and showing less cytoplasmic areas (Fig 6C).

## Discussion

Shortage of liver for organ transplantation to treat end-stage liver disease and lengthy waiting lists of organ recipients explains the importance of developing functional liver *ex vivo* through tissue engineering [17]. The intention to reduce animal experiments is of high priority and in addition, animal models can never fully reflect the human cell interactions. Conventional *in vitro* systems often lack the necessary cellular cross talk and cells in these systems have often lost major properties of the corresponding primary cells, which profoundly limits their possible applications.

In this manuscript we demonstrate that organoid structures with some similarities to adult liver tissue can be generated *in vitro* using differentiated adult human cells. Similar structures were generated earlier by Takebe et al. [5] using endodermal cells derived from induced pluripotent stem (iPS) cells combined with HUVECs and mesenchymal stem cells (MSCs). The authors demonstrated that MSCs play a pivotal role for this three-dimensional structure formation. MSCs are multipotent, self-renewing, adult stem cells, which can differentiate into hepatocytes (reviewed in [18]). Such differentiation can be induced by addition of growth factors like hepatocyte growth factor (HGF) or basic fibroblast growth factor (bFGF) [19] or by co-culturing the cells with hepatocytes [20], [21].

Because MSC-derived hepatocytes acquire the ability to express hepatocyte marker genes like albumin it would be difficult to distinguish between the original hepatocytes and MSC-derived hepatocytes in our 10-day organoids, therefore we cannot exclude that at least some of the hepatocytes in the organoids originated from MSCs. However, direct differentiation of MSCs into hepatocytes might not represent the major mechanism how MSCs support survival of liver tissue as it has been shown that intravenous bolus of conditioned medium from MSC can already provide a significant survival benefit in rats undergoing fulminant liver failure [22]. Therefore the possible hepatoprotective effect of MSCs seems to be mainly based on the secretion of factors which on the one side may modulate cells of the immune system (which are not present in our setup) but also exert direct anti-apoptotic effects on hepatocytes [23]. The exact nature of these MSC-derived factors that might be essential for organoid formation are yet to be explored. However, Takebe et al. have already described that application of inhibitors against bone morphogenetic proteins (BMPs) or fibroblast growth factor (FGF) effectively prevented the structure formation in their setting [5].

The data presented here demonstrate that differentiated adult upcyte^®^ human cells can be used for the formation of three-dimensional hepatic structures *in vitro* which are viable and functional up to a period of 10 days in culture (longer culture times are currently under investigation). Such structures resemble the three-dimensional architecture of the *in vivo* human liver better than conventional monolayer cultures consisting of hepatoma cell lines or isolated primary cells. Also, availability of these primary cells from human sources is rather sporadic and not accessible for everybody. Apart from this, primary cells show a highly unstable phenotype in culture and produce very variable results due to donor differences and/or varying effectiveness of the isolation process itself. Upcyte^®^ cell strains which exhibit primary cell phenotypes can be obtained in large amounts from the same donor which meets the demand for comprehensive sets of experiments. Due to the standardized protocol, results obtained with this system are highly reproducible. The use of such organoid liver structures generated *in vitro* is therefore suitable for a large range of applications such as the testing of subchronic or chronic toxicity caused by slow metabolized compounds and in addition it may help to reduce the number of animal experiments required for therapeutic drug development in the future.

Our results show that hepatocytes within such organoids express epithelial membrane markers like E-Cadherin and ZO–1, implying that compared to monolayer cultures they retain a more polarized morphology. We also show that the cells express albumin and produce several drug metabolizing enzymes to a degree that is comparable to adult human liver. This demonstrates that the liver organoids represent a 3D set up which is most likely superior to monolayer cultures of primary hepatocytes where the cells rapidly lose polarity and enzymatic functionality. Nevertheless, the cells within the organoids showed very high proliferative activity which might point to the conclusion that after 10 days the structures are still developing and that they did not (yet) reach a fully differentiated status.

Ongoing experiments aim at further optimizing the parameters for long term culture and differentiation of liver organoids. Additionally, integration of further liver cell types like Kupffer cells and hepatic stellate cells is planned in order to generate structures which resemble even more closely the *in vivo* conditions.

## Supporting Information

S1 Fig Photographs of liver organoids after 24 or 48h. Liver organoid preparation can be down-scaled from 24-well format to 48- or 96-well plate formats.
(TIF)

## References

[1] Schanz S, Schmalzing M, Guenova E, Metzler G, Ulmer A, Kötter I, et al (2012) Interstitial granulomatous dermatitis with arthritis responding to tocilizumab. Arch Dermatol 148: 17–20. 10.1001/archdermatol.2011.341 22250229

[2] Matsusaki M, Case CP, Akashi M (2014) Three-dimensional cell culture technique and pathophysiology. Adv Drug Deliv Rev 74: 95–103. 10.1016/j.addr.2014.01.003 24462454

[3] Cienfuegos JA, Rotellar F, Baixauli J, Martinez-Regueira F, Pardo F, Hernández-Lizoáin JL (2014) Liver regeneration–-the best kept secret. A model of tissue injury response. Rev Esp Enferm Dig 106: 171–194. 25007016

[4] Kurinna S, Barton MC (2011) Cascades of transcription regulation during liver regeneration. Int J Biochem Cell Biol 43: 189–197. 10.1016/j.biocel.2010.03.013 20307684 PMC2923255

[5] Takebe T, Sekine K, Enomura M, Koike H, Kimura M, Ogaeri T, et al (2013) Vascularized and functional human liver from an iPSC-derived organ bud transplant. Nature 499: 481–484. 10.1038/nature12271 23823721

[6] Sorensen KK, McCourt P, Berg T, Crossley C, Le Couteur D, Wake K, et al (2012) The scavenger endothelial cell: a new player in homeostasis and immunity. Am J Physiol Regul Integr Comp Physiol 303: R1217–1230. 10.1152/ajpregu.00686.2011 23076875

[7] Norenberg A, Heinz S, Scheller K, Hewitt NJ, Braspenning J, Ott M (2013) Optimization of upcyte® human hepatocytes for the in vitro micronucleus assay. Mutat Res 758: 69–79. 10.1016/j.mrgentox.2013.09.008 24140631

[8] Burkard A, Dahn C, Heinz S, Zutavern A, Sonntag-Buck V, Maltman D, et al (2012) Generation of proliferating human hepatocytes using Upcyte® technology: characterisation and applications in induction and cytotoxicity assays. Xenobiotica 42: 939–956. 10.3109/00498254.2012.675093 22524704

[9] Levy G, Bomze D, Heinz S, Ramachandran SD, Noerenberg A, Shibolet O, et al (2015) Genetic Induction of Metabolically Functional, Polarized Cultures of Proliferating Human Hepatocytes. Nature Biotechnology, *in press*. 10.1038/nbt.3377 26501953

[10] Ramachandran SD, Vivares A, Klieber S, Hewitt NJ, Muenst B, Heinz S, et al (2015) Applicability of second-generation upcyte® human hepatocytes for use in CYP inhibition and induction studies. Pharma Res Per 3(5). 2015; *in press*. 10.1002/prp2.161 PMC4618636 26516577

[11] Ghafoory S, Breitkopf-Heinlein K, Li Q, Dzieran J, Scholl C, Dooley S, et al (2012) A fast and efficient polymerase chain reaction-based method for the preparation of in situ hybridization probes. Histopathology 61: 306–313. 10.1111/j.1365-2559.2012.04237.x 22458731

[12] Ghafoory S, Breitkopf-Heinlein K, Li Q, Scholl C, Dooley S, Wölfl S. (2013) Zonation of nitrogen and glucose metabolism gene expression upon acute liver damage in mouse. PLoS One 8: e78262 10.1371/journal.pone.0078262 24147127 PMC3798318

[13] Zeitler P, Pahnke J, Marx A (2004) Expression of stromelysin–1 (MMP–3), gelatinase B (MMP–9), and plasminogen activator system during fetal calvarial development. Histopathology 44: 360–366. 15049902 10.1111/j.1365-2559.2004.01854.x

[14] Hewitt NJ, Lechon MJ, Houston JB, Hallifax D, Brown HS, Maurel P, et al (2007) Primary hepatocytes: current understanding of the regulation of metabolic enzymes and transporter proteins, and pharmaceutical practice for the use of hepatocytes in metabolism, enzyme induction, transporter, clearance, and hepatotoxicity studies. Drug Metab Rev 39: 159–234. 17364884 10.1080/03602530601093489

[15] Zanger UM, Klein K (2013) Pharmacogenetics of cytochrome P450 2B6 (CYP2B6): advances on polymorphisms, mechanisms, and clinical relevance. Front Genet 4: 24 10.3389/fgene.2013.00024 23467454 PMC3588594

[16] Mendieta-Wejebe JE, Correa-Basurto J, Garcia-Segovia EM, Ceballos-Cancino G, Rosales-Hernandez MC (2011) Molecular Modeling Used to Evaluate CYP2C9-Dependent Metabolism: Homology Modeling, Molecular Dynamics and Docking Simulations. Curr Drug Metab. 10.2174/138920011795713670 21486213

[17] Rai R (2013) Liver transplantatation- an overview. Indian J Surg 75: 185–191. 10.1007/s12262-012-0643-0 PMC3689386 24426424

[18] Volarevic V, Nurkovic J, Arsenijevic N, Stojkovic M (2014) Concise review: Therapeutic potential of mesenchymal stem cells for the treatment of acute liver failure and cirrhosis. Stem Cells. 10.1002/stem.1818 25154380

[19] Shu SN, Wei L, Wang JH, Zhan YT, Chen HS, Wang Y. (2004) Hepatic differentiation capability of rat bone marrow-derived mesenchymal stem cells and hematopoietic stem cells. World J Gastroenterol 10: 2818–2822. 15334677 10.3748/wjg.v10.i19.2818 PMC4572109

[20] Lange C, Bassler P, Lioznov MV, Bruns H, Kluth D, Zander AR, et al (2005) Liver-specific gene expression in mesenchymal stem cells is induced by liver cells. World J Gastroenterol 11: 4497–4504. 16052678 10.3748/wjg.v11.i29.4497 PMC4398698

[21] Luk JM, Wang PP, Lee CK, Wang JH, Fan ST (2005) Hepatic potential of bone marrow stromal cells: development of in vitro co-culture and intra-portal transplantation models. J Immunol Methods 305: 39–47. 16150456 10.1016/j.jim.2005.07.006

[22] Parekkadan B, van Poll D, Suganuma K, Carter EA, Berthiaume F, Tilles AW, et al (2007) Mesenchymal stem cell-derived molecules reverse fulminant hepatic failure. PLoS One 2: e941 17895982 10.1371/journal.pone.0000941 PMC1978513

[23] Xagorari A, Siotou E, Yiangou M, Tsolaki E, Bougiouklis D, Sakkas L, et al (2013) Protective effect of mesenchymal stem cell-conditioned medium on hepatic cell apoptosis after acute liver injury. Int J Clin Exp Pathol 6: 831–840. 23638214 PMC3638093

