# Supplementary figures and images for: In Vitro Generation of Functional Liver Organoid-Like Structures Using Adult Human Cells
Source: PLoS One. 2015 Oct 21;10(10):e0139345. doi: 10.1371/journal.pone.0139345 (PMC4619350; doi:10.1371/journal.pone.0139345)

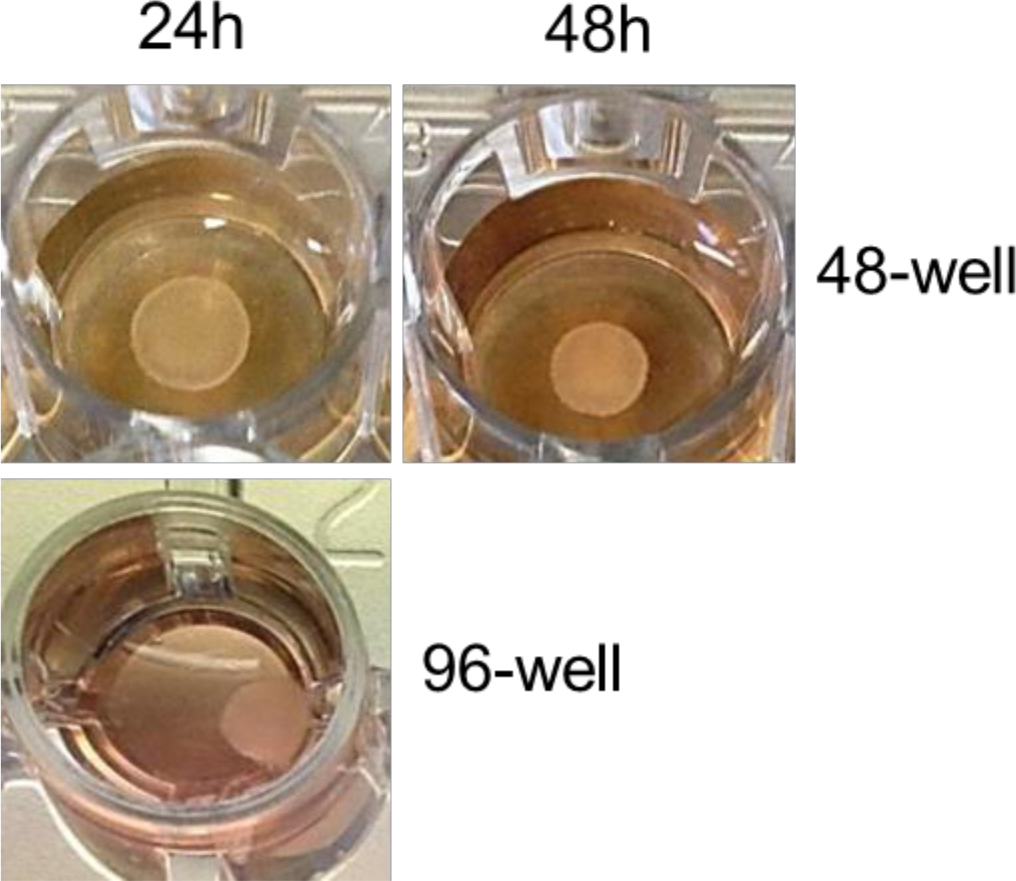

Supplement: S1 Fig — Liver organoid preparation can be down-scaled from 24-well format to 48- or 96-well plate formats. (TIF) [file pone.0139345.s001.tif]
